# Dietary Antioxidants and the Mitochondrial Quality Control: Their Potential Roles in Parkinson’s Disease Treatment

**DOI:** 10.3390/antiox9111056

**Published:** 2020-10-28

**Authors:** Davin Lee, Min Gu Jo, Seung Yeon Kim, Chang Geon Chung, Sung Bae Lee

**Affiliations:** 1 Department of Brain & Cognitive Sciences, DGIST, Daegu 42988, Korea; cloudavin@dgist.ac.kr (D.L.); whalsrnrc@dgist.ac.kr (M.G.J.); syeon1111@dgist.ac.kr (S.Y.K.); 2 Protein Dynamics-Based Proteotoxicity Control Laboratory, Basic Research Lab, DGIST, Daegu 42988, Korea

**Keywords:** dietary antioxidants, mitochondria, reactive oxygen species, neurodegenerative disease, Parkinson’s disease, mitochondrial quality control

## Abstract

Advances in medicine and dietary standards over recent decades have remarkably increased human life expectancy. Unfortunately, the chance of developing age-related diseases, including neurodegenerative diseases (NDDs), increases with increased life expectancy. High metabolic demands of neurons are met by mitochondria, damage of which is thought to contribute to the development of many NDDs including Parkinson’s disease (PD). Mitochondrial damage is closely associated with the abnormal production of reactive oxygen species (ROS), which are widely known to be toxic in various cellular environments, including NDD contexts. Thus, ways to prevent or slow mitochondrial dysfunction are needed for the treatment of these NDDs. In this review, we first detail how ROS are associated with mitochondrial dysfunction and review the cellular mechanisms, such as the mitochondrial quality control (MQC) system, by which neurons defend against both abnormal production of ROS and the subsequent accumulation of damaged mitochondria. We next highlight previous studies that link mitochondrial dysfunction with PD and how dietary antioxidants might provide reinforcement of the MQC system. Finally, we discuss how aging plays a role in mitochondrial dysfunction and PD before considering how healthy aging through proper diet and exercise may be salutary.

## 1. Introduction

As life expectancy continues to increase, the elderly population and their risk for age-related diseases also grow. While aging of the body is accompanied by complications in most cell types, neurons are particularly vulnerable to cellular aging as evidenced by the significant decline in sensory, motor, and cognitive functions with time [1,2]. This is partly due to the fact that despite its relatively small mass (approximately 2% of body mass), the brain consumes about 20% of the body’s cellular energy and produces a substantial amount of metabolic waste [3]. If not cleared efficiently, these waste products accumulate and lead to serious neuronal impairments, which may gradually disintegrate neural integrity, result in neuronal aging, and cause a variety of neurodegenerative diseases (NDDs). Another aspect of neuronal vulnerability to aging is the fact that degenerated neurons are virtually irreplaceable; the replacement of neurons through either neurogenesis or the bypass of degenerated neurons through neural rewiring is very limited. It is therefore imperative to seek ways to protect neurons before they die—by preventing or overcoming age-related neuronal damage—to reduce the chances of developing age-related diseases and the societal burden that comes with a growing elderly population with NDDs.

Due to their large metabolic demand, neurons have a high concentration of functional mitochondria to supply cellular energy [4]. As such, neurons are particularly sensitive to mitochondrial damage [5,6]. Mitochondrial damage is largely contributed by the unmonitored production and inadequate clearance of mitochondrial reactive oxygen species (ROS), the byproducts of mitochondria-dependent energy production. As a result, neurons bear the burden of maintaining a functionally intact mitochondrial pool protected from ROS-induced mitochondrial damage. The surveillance of mitochondrial quality and turnover of defective mitochondria are accomplished through a series of cellular processes, collectively called the mitochondrial quality control (MQC) process [7]. These are largely divided based on mitochondrial damage: processes that prevent mitochondrial damage and processes that turn over damaged mitochondria.

At an early stage of mitochondrial damage, the MQC prevents further damage by recognizing and selectively removing non-assembled and misfolded proteins using ATP-dependent proteases and the ubiquitin proteasome system (UPS) [8]. Another way to minimize mitochondrial damage at an early stage is to dilute the damage by inducing fusion of a damaged mitochondrion with a healthy mitochondrion via mitofusins and OPA1 [9]. Alternatively, when the damage is considerable, the damaged portion of the mitochondrion is asymmetrically distributed or aggregated. Then, through the process of DRP1-mediated fission, two mitochondria are produced: one that is mostly damaged and another that is mostly healthy. PINK1/Parkin-mediated mitophagy [10] subsequently eliminates only the damaged mitochondrion. Thus, mitophagy appears to be intimately linked to mitochondrial fission and fusion processes that are critical for mitochondrial functions, including regulation of Ca^2+^ flux [11,12].

Although intrinsic cellular programs/enzymes (such as peroxiredoxin (Prx) and superoxide dismutase (SOD)) help reduce mitochondrial damage by neutralizing intracellular ROS in neurons [13], the abnormal production of ROS may still occur by overriding the capacity of the intrinsic defense system and result in mitochondrial damage. If damaged mitochondria are left unchecked, they may further amplify ROS-associated stress by decreasing the expression of proteins critical for electron transport, leading to a vicious cycle of ROS production and mitochondrial dysregulation [14]. Thus, mitochondrial homeostasis, which is crucial for neuronal homeostasis and survival, is intricately balanced through the surveillance of mitochondrial damage, removal of damaged mitochondria, and biogenesis of healthy mitochondria based on cellular needs.

When this balance is disturbed—either through the abnormal production of ROS or specific genetic mutations—the damaged mitochondria accumulate and may contribute to the development of various age-related NDDs such as Parkinson’s disease (PD) [15]. PD, the second most common NDD, is caused by dopaminergic neuronal loss in the substantia nigra. Overwhelming evidence indicates that the dysfunction associated with MQC and the unmonitored production of ROS are central factors associated with PD pathophysiology [16,17]. For example, PINK1 mutations, which are the second most common cause of autosomal recessive early-onset PD, lead to an impairment of various aspects of mitochondrial biology, including mitophagy and mitochondrial fission [17,18,19]. In addition, phenotypes consistent with sporadic PD can be induced by a number of inhibitors of mitochondrial function (including rotenone, 1-methyl-4-phenyl-1,2,3,6-tetrahydropyridine (MPTP), paraquat, nitric oxide, the dopamine metabolite aminochrome, and others), which drive high levels of ROS production [16]. It still remains elusive, however, whether and how mitochondria located in the distal dendritic or axonal area, aside from the well-characterized perinuclear mitochondria, contribute to PD, given the profound loss of dendritic spines and the remodeling of axospinous glutamatergic synapses seen in PD patients [20]. The importance of mitochondrial health in most NDDs, and particularly PD, has prompted many researchers to search for factors that influence and/or promote the optimal operation of MQC processes. In this review, we present dietary antioxidants as such factors, particularly in the PD context. In line with this, previous studies support the idea that dietary antioxidants may have beneficial effects, at least in certain types of NDDs, including PD [21], amyotrophic lateral sclerosis [22], and Alzheimer’s disease [23].

Here, we review how mitochondrial ROS affect MQC processes, propose ways in which antioxidants may be utilized to raise the overall capacity and/or power of MQC processes for the amelioration of PD symptoms, discuss how aging impacts mitochondrial dysfunction and PD, and briefly consider the potential benefits of proper diet and exercise.

## 2. The Physiological Role of ROS and the Pathological Consequence of Excessive ROS-Induced Toxicity

ROS is a collective term referring to chemically reactive oxygen derivatives that are byproducts of normal metabolic processes. Generally, there are two groups of ROS: radical ROS which contain at least one unpaired electron, and non-radical ROS which do not. The radical ROS group (which includes superoxide (O_2_**^˙^**^−^), hydroxyl (**^˙^**OH), alkoxy radical (RO**^˙^**), and peroxyl radical (ROO**^˙^**)) is characterized by its tendency to donate or obtain unpaired electrons to attain stability. The non-radical ROS group (which includes peroxide (H_2_O_2_), hypochlorous acid (HOCl), and singlet oxygen (^1^O_2_)) does not contain free radicals, though non-radical ROS can easily lead to the production of free radicals in vivo [24]. Depending on their location and build-up, both radical and non-radical ROS can be either detrimental reactive agents that bring pathological complications to the given cellular environment or be used as mediators/inducers regulating various cellular activities. Although ROS were traditionally thought of as unavoidable toxic byproducts of aerobic metabolism, a recent, more nuanced perspective suggests that maintaining basal levels of ROS in cells is essential for the life of an organism [25,26]. This is partly due to the discovery of the endogenous role of ROS in cell signaling cascades, programmed cell death, necrosis, and the induction or suppression of many important genes [27,28,29]. Additionally, ROS are known to be tightly associated with the body’s inflammatory processes by regulating the expression of nuclear factor-kB [30]. Despite the important roles of ROS in cells, their abnormal production can substantially deteriorate the cellular condition by disrupting intracellular redox homeostasis and abnormally oxidizing various biological macromolecules, such as lipids, proteins, carbohydrates, and DNA [31]. For example, the excessive production of **^˙^**OH can lead to single- and double-strand breaks in DNA, peroxidation of lipids, and fragmentation of various proteins [32,33,34]. The toxicity associated with the excessive production of ROS led researchers to investigate the biological mechanisms behind the generation of oxidative agents.

**Figure 1:**
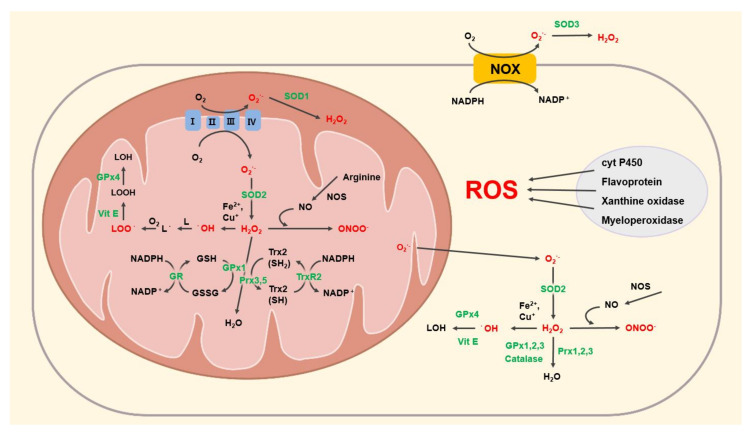
A schematic illustration showing the generation of ROS in mitochondria and other cellular regions. The red-colored letters refer to ROS, while the green-colored letters refer to enzymes that are associated with the reduction of ROS. Briefly, in mitochondria, the electron transport chain (ETC) produces and releases O_2_**^˙^**^−^ into the intermembrane space and the matrix. O_2_**^˙^**^−^ is then converted to H_2_O_2_ by superoxide dismutase (SOD). H_2_O_2_ is converted to water by the glutathione peroxidase (GPx) or peroxiredoxin (Prx) pathways. The Fenton reaction between Fe^2+^ and H_2_O_2_ yields **^˙^**OH, which initiates lipid peroxidation. Through vitamin E (Vit E) and GPx4, LOO**^˙^** is converted to LOH. NOS synthesizes NO from arginine. NO then can react with H_2_O_2_ to produce ONOO^−^. This same process also occurs in the cytoplasm. ROS can also be produced by other enzymes such as NOX, cytochrome P450 (cyt P450), Flavoprotein, Xanthine oxidase, and Myeloperoxidase. Roman numerals (I, II, III, and IV) refer to mitochondrial ETC complexes.

ROS are primarily produced in two main hubs: the NADPH oxidase (NOX) pathway and the mitochondrial electron transport chain (ETC) (Figure 1). The NOX family of enzymes comprises membrane-bound proteins that typically localize in the plasma membrane, though some reports indicate that they are also found in various membrane-bound organelles including endosomes, lysosomes, endoplasmic reticulum (ER), mitochondria, and the nuclei [35]. Although there are seven NOX homologues in humans (Nox1-5 and Duox1-2) with distinctive expression patterns, interacting partners, and types of ROS generated, all NOX proteins ultimately generate O_2_**^˙^**^−^ and H_2_O_2_ during the conversion of NADPH to NADP^+^ + H^+^ [36]. The mitochondria, on the other hand, produce O_2_**^˙^**^−^ during the process of oxidative phosphorylation associated with the ETC [37]. Although the ETC is generally responsible for the production of ATP, it can also produce ROS generated in mitochondria via the leakage of electrons during the transfer of electrons from ETC complexes I to III [38]. These leaked electrons react with oxygen to form O_2_**^˙^**^−^, which is converted to H_2_O_2_ by SOD [39]. Besides NOXs and ETC, other enzymatic processes such as those mediated by xanthine oxidase, myeloperoxidase, and cytochrome P450 generate modest amounts of ROS in various cellular regions. Reports indicate that ER produces ROS during the disulfide bond modification by flavoproteins [40], while peroxisome produces ROS during the transformation of hypoxanthine to uric acid by xanthine oxidase in purine metabolism processes [41]. Lysosome is associated with the production of HOCl through the enzymatic activity of myeloperoxidase to remove exogenous pathogens engulfed by phagocytosis [42]. Another example is cytochrome P450 which produces O_2_**^˙^**^−^ and H_2_O_2_ through the catalytic cycle in several cellular regions, including the ER, nucleus, mitochondria, and cytoplasm [40]. Although all of these processes contribute to ROS production in cells, mitochondria are likely the predominant contributor of ROS [43]. Considering that each neuron in the substantia nigra is estimated to have ~two million mitochondria [4], the leakage of electrons from ETC—and thus ROS—could be substantially higher in a neuron than in a typical cell with only thousands of mitochondria.

When ROS are excessively produced or inappropriately localized within the cell, they abnormally oxidize biological substances such as DNA, RNA, proteins, lipids, and metabolites, which leads to various complications [31]. Therefore, cells must protect themselves from ROS to maintain cellular homeostasis. As one of the main regions responsible for the production of ROS, mitochondria are notably vulnerable to excessive ROS [44]. When mitochondrial ROS handling ability is negatively impacted by either the abnormal increase in ROS production or the decrease in mitochondrial ROS buffering capacity, various mitochondrial dysfunctions occur. These include mutations in mitochondrial DNA (mtDNA) and alterations in mitochondrial physiology such as membrane permeability and Ca^2+^ homeostasis [14,44,45,46]. Of note, the perinuclear region of a neuron accounts for only a small proportion of the neuronal area. As eloquently described by Misgeld and Schwarz, a single neuron of the human substantia nigra is estimated to contain approximately 2 million mitochondria [4]. Given how mitochondria are highly concentrated in both perinuclear and distal dendritic/axonal regions, neurons are particularly vulnerable to both excessive ROS production within the soma and in distal neuronal regions. When ROS damage the mitochondria at a certain level, the damaged mitochondria can further enhance the production of ROS by decreased expression of proteins associated with ETC, thereby leading to a vicious cycle of ROS production [14]. Given the importance of maintaining a healthy mitochondrial pool, we will next highlight the intake of dietary antioxidants as a way by which the harmful effect of ROS may be mitigated to achieve cellular homeostasis.

## 3. Defense Mechanisms against ROS

To defend against toxic levels of ROS, cells have intrinsic mechanisms involving a direct reduction of ROS through the enzymatic activities of various proteins including SOD, glutathione (GSH), and thioredoxin (Trx) (Figure 1 and Table 1), whose functions are complemented by the action of dietary antioxidants (Table 2). Additionally, cells employ processes that adjust the production of ROS in the mitochondria by properly maintaining a healthy mitochondrial pool.

Here, we provide a comprehensive summary of intrinsic enzymes that are involved in the reduction of ROS, as shown in Table 1. The SODs are major antioxidant defense enzymes that convert O_2_**^˙^**^−^ to H_2_O_2_. Reports indicate that SODs protect against oxidative stress in various cellular regions, including mitochondria, depending on the type of SOD [39]. Additionally, H_2_O_2_ can either be further detoxified into H_2_O via the glutathione peroxidase (GPx) and Prx pathways [47,48] or be transformed into **^˙^**OH by Fenton reaction mediated by Fe^2+^ and Cu^+^ [49]. **^˙^**OH then react with membranous lipids to produce lipid peroxy radical LOO**^˙^** [50], which is then sequentially detoxified by vitamin E and GPx [51]. Therefore, we suggest that the gradual decrease in the capacity of ROS defense mechanisms and/or accumulated damage derived from ROS leaked from these defenses may be associated with aging and the pathogenesis of various age-related diseases.

Given that the maximum capacity of the intrinsic defense mechanism is limited and gradually declines with time [52,53], the compensatory role of dietary antioxidants may be utilized in the current aging society to slow down biological aging and prevent certain age-related diseases. Antioxidants are defined as chemical compounds that inhibit oxidation. Dietary antioxidants can be classified into various groups such as vitamins, bioflavonoids, carotenoids, and hydroxycinnamates. Many vegetables and fruits provide ample sources of antioxidants (Table 2).

Antioxidants may neutralize the damage associated with free radicals by (1) directly reacting with free radicals as scavengers or (2) acting indirectly through either inhibition of ROS production or enhancement of the function of intrinsic enzymes that defend against ROS [54]. Vitamins A, C, and E are direct antioxidants and among the most potent reducing agents and scavengers of free radicals in biological systems. These vitamins are especially vital antioxidants that protect LDL oxidation [55,56,57]. Carotenoids (the organic pigments that are involved in the coloration of plants and algae) are another direct antioxidant that interact with free radicals and singlet oxygen through the polyene backbone structural element (consisting of C = C bonds which allow them to exert their antioxidant properties) [58]. Another group of direct antioxidants, hydroxycinnamates, has been shown to protect LDL from oxidation by free radical scavenging [59]. Examples of indirect antioxidants include polyphenols or polyphenol-derived compounds such as catechin, resveratrol, flavonoids, and isoflavone; these are hormetic effectors that indirectly reduce ROS in vivo by activating the redox- sensitive Keap1-Nrf2-ARE regulatory pathway [60]. Research points to the fact that supplementation of antioxidants can result in the reduction in ROS toxicity, potentially hinting at a therapeutic treatment for diseases associated with ROS, however, the results of clinical trials for antioxidant-related drugs as a sole treatment remain inconclusive. We will elaborate on the apparently limited effects of antioxidant-related drugs in human clinical trials in the Discussion section below.

One approach used by cells as a defense against ROS is to employ the site-specific mechanisms such as MQC that help adjust ROS production in mitochondria, the key ROS-producing site. Maintenance of a healthy mitochondrial pool by the MQC system can be important for the overall defense against ROS since mitochondria themselves are thought to actively buffer/stabilize ROS levels [61,62]. However, as the MQC system is expected to imperfectly safeguard mitochondria from ROS-associated damage, the proportion of damaged mitochondria is likely to increase with age. To complement the imperfect protection of mitochondria by the MQC system, appropriate care to control ROS through dietary antioxidants appears to be crucial for both healthy aging and the prevention of certain age-related diseases such as PD.

## 4. The Potentially Beneficial Effects of Dietary Antioxidants in Parkinson’s Disease Mediated through the Reinforcement of the Mitochondrial Quality Control System

PD, having been causally linked to the oxidative damage in the dopaminergic neurons of the substantia nigra [63], is a representative NDD associated with mitochondrial dysfunction derived from either excessive ROS toxicity or the aberration of the MQC system. PD is a progressive neurological disorder, major clinical symptoms of which include tremor, rigidity, and bradykinesia. Etiologically, PD is known to be caused by both environmental (90–95%) and genetic (5–10%) factors, with overwhelming evidence suggesting that mitochondrial damage is a central factor associated with its pathophysiology [64]. The current model for PD pathogenesis highlights the role of damaged mitochondria, which aberrantly produce ROS that contribute to the deterioration of most, if not all, normal cellular processes in neurons; this ultimately increases the neuronal vulnerability to neurodegeneration. Many causal factors associated with PD have been identified to date, such as the metabolism of dopamine, the impairment of mitophagy, dysfunction of ETC, induction of aberrant neuroinflammation, and aging, most of which are associated with mitochondrial dysfunction [65,66]. In the current model for PD pathogenesis, ROS play a dual role as a causal factor for mitochondrial damage and a mediator of neuronal toxicity.

The link between oxidative stress and dopaminergic neuronal degeneration in PD is further supported by the fact that the treatment with chemicals (such as MPTP, rotenone, and 6-hydroxydopamine (6-OHDA)) that impair the ETC and induce excessive ROS production mimics the PD-like motor symptoms in animal models [67,68,69,70]. For example, rotenone, a well-known inhibitor of ETC I, prevents the transport of electrons from ETC I to ubiquinone by blocking the ubiquinone binding site. The prevention of electron transport by rotenone leads to the formation of ROS, particularly O_2_**^˙^**^−^, in the mitochondrial matrix [71]. In line with this, it is well known that some of these chemicals are among the causal factors of environmentally induced PD [72]. In addition, some of the known genetic factors for PD, including Parkin (Park2) and PINK1 (Park6), cooperatively play a central role in the MQC system by regulating the mitophagy of damaged mitochondria [73]. The morphological changes of mitochondria were first reported in fly models for Parkin or PINK1, followed by the discovery of impaired mitochondrial dynamics and mitophagy in association with PD [18,19,64]. Further mechanistic studies revealed that in healthy mitochondria, the mitochondrial kinase PINK1 undergoes degradation mediated by mitochondria-specific proteases such as PARL and OMA1 [74,75], whereas PINK1 accumulates on the outer membrane of damaged mitochondria. On the outer membrane, PINK1 recruits the E3 ligase Parkin, which induces degradation of mitochondrial surface proteins such as VDAC and mitofusin, required for mitophagy via ubiquitination [76,77,78]. The resulting accumulation of damaged mitochondria by impaired mitophagy (derived from genetic mutations of PINK1 and Parkin) leads to further exacerbation of ROS production and may eventually result in neuronal insult. Thus, proper handling of ROS appears to be crucial for the prevention and/or amelioration of PD.

Since the PD disease condition reflects the failure of the intrinsic MQC system to properly handle ROS and maintain a healthy mitochondrial pool, a supply of exogenous antioxidants that increase the power and capacity of the MQC system may be beneficial to neurons. Dietary antioxidants can reduce ROS, which primarily damage mitochondria, and thus reduce the chance of damaged mitochondria accumulation and resultant PD. Dietary antioxidants can also reduce the overall ROS toxicity in a given cell and by doing so interrupt disease pathogenesis. Through these two mechanisms, dietary antioxidants may halt the vicious cycle that leads to the abnormal production of ROS. In addition to the canonical mechanisms by which antioxidants reduce ROS levels, research has shown that antioxidants may improve MQC processes, thereby indirectly contributing to ROS homeostasis. Creatine, for example, can not only serve as a direct antioxidant [79], but can also improve mitochondrial function by enhancing mitochondrial biogenesis and preventing a decrease in mitochondrial membrane potential and ATP content [80]. Additionally, creatine is implicated in protecting mitochondria from mtDNA damage induced by oxidative stress [81]. Furthermore, flavonoid derivatives are strong antioxidants that can ameliorate mitochondrial dysfunction by promoting mitochondrial membrane potential and reinforcing ETC IV, ultimately leading to the reduction in mitochondrial ROS production [82].

Supporting the possibility that dietary antioxidant treatment can be beneficial to diseased neurons in PD, previous studies showed that treatment with dietary antioxidants resulted in improved mitochondrial health and neuronal function in in vitro PD models and improvements in animal behavior in in vivo PD models. For example, baicalein, a major bioactive flavone, was reported to attenuate muscle tremor and increase the number of tyrosine hydroxylase (TH) neurons in rotenone-induced parkinsonian rats. Baicalein seems to be able to ameliorate the PD phenotype in a SH-SY5Y PD cell model by improving mitochondrial function and biogenesis through activation of the CREB/GSK-3β/PGC-1α pathway [83]. Additionally, hesperidin, a citrus fruit flavanol, showed neuroprotective effects in a rotenone-induced neuroblastoma cell model of PD by exerting its antioxidant effects to support the maintenance of mitochondrial function [84]. Furthermore, hesperidin is reported to ameliorate both neuronal and behavioral parameters in a 6-OHDA-injected aged PD mouse model [85]. Consistently, a few human cohort studies showed that the supplementation of dietary antioxidants is associated with reduced risk of PD and amelioration of PD symptoms. In a study that prospectively examined the effects of dietary intake of antioxidants in 84,774 participants, vitamin E and ß-carotene were shown to be synergistically associated with a lower risk of PD [86]. Another report indicates that creatine seems to delay the functional decline in cognitive abilities seen in PD patients [87]. However, several other cohort studies showed inconsistent results related to the beneficial effects of dietary antioxidants on PD symptoms [88,89,90]. For example, a study that treated 60 PD patients with creatine (10 g per day) for several years did not improve the clinical outcome of patients [89]. In addition, a meta-analysis that pooled eight studies that investigated the relationship between antioxidants (such as vitamin A or carotenoids) and the progression of PD showed no statistically significant relationship [90]. We speculate that this inconsistency may be partly associated with the technical limitations in optimizing the delivery and effectiveness of dietary antioxidants required to override PD-linked mitochondrial damage in neurons (see Discussion for details on these limitations). Taken together, these data show that although dietary antioxidants show beneficial, experimentally validated effects in both in vitro and in vivo PD models, further studies are required to optimize their delivery and functionality in neurons.

## 5. The Impact of Aging on Mitochondrial Dysfunction and Parkinson’s Disease, and the Potentially Beneficial Effects of Proper Diet and Exercise

Aging is strongly correlated with not only PD, but also with mitochondrial dysfunction. As mitochondria age, ROS-induced mutations in mtDNA accumulate at 10 times the rate of mutations in nuclear DNA [91]. These mutations eventually produce faulty ETC proteins, which trigger ROS production. This vicious cycle of ROS-induced toxicity and ROS production may contribute to the development of NDDs. Although chronological aging allows ROS to accumulate in mitochondria, the mitochondrial dysfunction that arises from ROS accumulation expedites biological aging. This means that although chronological and biological aging are highly correlated, this correlation can be disrupted by intervening with ROS accumulation in mitochondria.

Apart from genetics, aging is one of the best predictors of PD. The percentage of dopaminergic neuronal loss in the substantia nigra has been reported to range from 4.7% [92] to 9.8% [93] per decade in humans. Furthermore, the number of pigmented neurons in the substantia nigra shows a strong negative correlation (r = −0.83) with age [93]. However, when biological aging is expedited via exposure to certain mitochondrial toxins, such as MPTP and paraquat, PD-like symptoms can develop at an earlier chronological age [94,95]. Based on these results, we propose that although chronological aging is a risk factor for PD, biological aging is likely the underlying modifier of disease development.

Although chronological aging cannot be altered, efforts can be made to curb biological aging through proper diet and exercise. In addition to the aforementioned protective effects of dietary antioxidants, drinking large amounts of tea or coffee may slow biological aging and provide some protection against PD [96,97]. Although earlier studies suggest that the beneficial effects of coffee may come mostly from caffeine, which antagonizes the adenosine A2A receptors [98,99], a therapeutic potential for polyphenols (such as chlorogenic acid) in coffee has recently been shown in mouse models [100,101] and an in vitro model of PD [102]. Indeed, polyphenols such as theaflavin in black tea [103] or (−)-epigallocatechin-3-gallate (EGCG) in green tea [104] have previously been suggested to provide protection against PD [97]. In addition, it might be a good idea to forgo a diet consisting of processed foods because they induce ROS toxicity [105,106]. Even drinking too much milk might increase the risk of PD [107], particularly in men [108,109]. The underlying factor in milk that increases the risk is still uncertain, but one study suggests that contamination of polychlorinated biphenyl and pesticides in milk might be a factor [109]. As for the high-fat diet, mouse studies indicate that it exacerbates neurodegeneration in a PD model [110,111], and human studies seem to suggest a link between animal fat consumption and PD [109,112]. However, other studies dispute this link [113,114]. Likewise, the link between a high carbohydrate diet and PD remains inconclusive [97,106]. Thus, although a proper diet may potentially be beneficial, the biggest problem at the moment is that there is no consensus on the definition of “proper diet”.

Exercise is largely accepted to be salutary. This is somewhat surprising, given the fact that exercise increases mitochondrial respiration [115] and thus ROS production. Nonetheless, numerous studies report the longevity of athletes [116,117], and exercise has been shown to mitigate some symptoms of NDDs including PD [118,119]. Studies suggest exercise and moderate amounts of ROS can lead to the up-regulation of brain-derived neurotrophic factor (BDNF) [120,121] and its receptor tyrosine-related kinase B (TrkB) [122,123], thereby promoting neuronal function and neurogenesis [124]. However, how much of the benefits from exercise actually come from exercise-induced ROS remains to be elucidated. Interestingly, for athletes who played contact sports such as soccer or American football, the cases of NDDs increased dramatically compared to the control population [125,126]. These data suggest that although exercise can promote healthy aging, contact sports that can physically damage neurons should likely be avoided.

## 6. Discussion

Oxidative stress and mitochondrial damage are widely presumed to constitute a pathologically relevant event in PD. In addition to PD, there are several NDDs (Table 3) that show a considerable association with mitochondrial damage and ROS, such as Charcot–Marie–Tooth disease type 2A, amyotrophic lateral sclerosis, Alzheimer’s disease, and Huntington’s disease [127,128,129,130,131]. Accordingly, treatment options for the amelioration of both oxidative stress and mitochondrial damage through the intake of dietary antioxidants have been extensively studied in both NDD animal models [131,132,133] as well as in in vitro models [131,132,133,134,135], resulting in a large number of putative treatment strategies. However, only a small number of human cohort studies have been successful in demonstrating any mitigating effects of antioxidant-based treatments on NDDs [136,137]. Why have antioxidants shown limited effects in clinical trials and how can antioxidant-based treatment be improved to help ameliorate age-related diseases? The limited effect of antioxidant-based treatment for NDDs in clinical trials has prompted some to suggest that oxidative stress may not be causally relevant to NDD progression [138]. This, however, contradicts many studies that have demonstrated the harmful effects of excessive ROS production on neuronal homeostasis [14,44,139,140]. The limited effect instead may stem from issues surrounding the delivery of dietary antioxidants to target neurons, insufficient knowledge about cellular ROS homeostasis, and short-termed human cohort studies related to NDD patients.

NDDs are typically characterized by the degeneration of disease-associated target neurons and the eventual loss of surrounding neurons. For dietary drugs to be effective in ameliorating NDDs, they must be adequately delivered to target neurons at a sufficiently effective concentration. The delivery of antioxidants to specific neuronal subpopulations is extremely difficult as the majority of NDDs require that the antioxidant enter neurons through the neuronal membranes. Additionally, for the antioxidants to be effective in reducing ROS in the mitochondria, a way to efficiently deliver antioxidants to the mitochondria is needed.

To meet this need, researchers have developed several antioxidant compounds that are specifically targeted to the mitochondria [184,185,186]. Among them is MitoQ, which is essentially a ubiquinone with a hydrophobic linker attached to a triphenylphosphonium (TPP) cation that targets mitochondria. Unfortunately, a clinical trial showed no significant effect of MitoQ in PD patients [187]. SKQ1, a plastoquinone derivative with a TPP cation attached, might be a better therapeutic option than MitoQ because SKQ1 has lower pro-oxidant activity and exerts antioxidant activity at a lower dose than MitoQ [188]. SKQ1 seems to readily cross the blood–brain barrier and localize in neuronal mitochondria in the brains of a rat model for AD [189]. Notably, SKQ1 treatment in rats significantly mitigated AD-like pathology and resulted in improved learning and memory. Likewise, SKQ1 treatment in a PD mouse model mitigated the decrease in dopamine quantity and partially blocked the loss of dopaminergic neurons in the substantia nigra [190]. Although no clinical trials have tested the efficacy of this molecule for the treatment of PD, a phase 2 clinical trial recently showed that topical treatment with SKQ1 significantly ameliorates dry eye symptoms in patients with Keratoconjunctivitis Sicca [191]. Like PD, dry eye symptoms are largely associated with oxidative stress [192]. These results suggest that SKQ1 can mitigate oxidative stress in humans and thus provide optimism that it may also be efficacious in treating PD.

Another aspect associated with the apparent ineffectiveness of antioxidants in human cohort studies may be linked to the ROS homeostasis maintained through the balancing of exogenous and endogenous antioxidants. ROS are essential in various cellular activities, suggesting that, though high levels are indeed toxic, maintaining the basal level of ROS is important [25,193]. Thus, any alterations that lead to an imbalance of intracellular ROS may trigger an endogenous defense response that attempts to restore ROS homeostasis. For example, many plant-derived antioxidants (such as resveratrol, isothiocyanate, catechins, or curcumin) are in fact phytochemical prooxidants [194,195,196] and believed to procure their antioxidant effects by promoting the endogenous Nrf2-mediated antioxidant response [195,197,198]. Similarly, the diet level of selenium, an essential trace element associated with antioxidant selenoproteins, seems to inversely correlate with various endogenous expressions of antioxidants and detoxifying enzymes [199]. Therefore, for antioxidants to be fully effective in ameliorating NDDs, a comprehensive understanding of the endogenous defense mechanisms that might weaken or otherwise negate dietary antioxidants is required. Additionally, developing ways to 1) maintain the basal level of ROS to protect normal cellular activities and 2) bypass endogenous defense responses is crucially needed.

Finally, we suggest that the ineffectiveness of antioxidants in human studies may also be due to the fact that the length of clinical trials have been too short to see whether antioxidants have beneficial effects in NDD patients over the long term. It may take several years, perhaps even decades, of gradual accumulation of degenerative insults before disease symptoms manifest. Therefore, expecting positive results from the intake of dietary antioxidants during the several years of clinical trials may be unrealistic. To understand whether antioxidants can slow or reverse disease progression, it may be important to identify potential NDD patients from familial cases and survey their disease incidence rate or progression based on the supplementation of dietary antioxidants over an extended period.

In this article, we provided our perspective view in support of the use of dietary antioxidants as a potential therapeutic option for NDDs associated with mitochondrial dysfunction. Given the previous studies as well as the limitations of dietary antioxidants compiled above, we propose that antioxidants may be beneficial as a supplementary option used along with disease-specific drugs. For this proposal to be viable, further studies that improve antioxidant-based treatment and research on the effect of the complementary use of antioxidant treatments are required.

## Figures and Tables

**Table 1:** A list and a brief description of the representative enzymatic antioxidants.

Enzymatic Antioxidant	Subtype	Locations	Associated Antioxidant Substrates	Associated Antioxidant Products
**Mn/Cu/Zn SOD (Superoxide dismutase)**	Cu/Zn SOD1	Mitochondrial intermembrane space, cytosol	O_2_˙^−^	H_2_O_2_
	Mn SOD2	Mitochondrial matrix		
	Cu/Zn SOD3	Extracellular space		
**Prx (Peroxiredoxin)**	Prx1	Nucleus, cytosol, plasma membrane	H_2_O_2_	H_2_O
	Prx2	Cytosol	H_2_O_2_	H_2_O
	Prx3	Mitochondrion, cytosol	H_2_O_2_	H_2_O
	Prx4	Endoplasmic reticulum, cytosol	H_2_O_2_	H_2_O
	Prx5	Mitochondrion, nucleus, peroxisome, cytosol	H_2_O_2_, ONOO^−^	H_2_O, ˙NO_2_
	Prx6	Cytosol	H_2_O_2_	H_2_O
**Trx (Thioredoxin)**	Trx1	Nucleus, cytosol, plasma membrane	H_2_O_2_	H_2_O
	Trx2	Mitochondrion		
**Grx (Glutaredoxin)**	Grx1	Nucleus, cytosol	H_2_O_2_	H_2_O
	Grx2	Nucleus, mitochondrion, cytosol		
	Grx3	Cytosol		
	Grx5	Mitochondrion		
**GPx (Glutathione peroxidase)**	GPx1	Mitochondrion, cytosol	H_2_O_2_, ONOO^−^	H_2_O, ˙NO_2_
	GPx2	Cytosol	H_2_O_2_	H_2_O
	GPx3	Cytosol	H_2_O_2_	H_2_O
	GPx4	Mitochondrion, plasma membrane	H_2_O_2_, ROO˙	H_2_O, ROH
	GPx6	Mitochondrion, nucleus, peroxisome, cytosol	H_2_O_2_	H_2_O
**CAT (Catalase)**		Peroxisome, cytosol	H_2_O_2_	H_2_O
**GR (Glutathione reductase)**		Mitochondrion, peroxisome, cytosol	H_2_O_2_, ROO˙	H_2_O, ROOH

**Table 2:** A list of representative dietary antioxidants and their canonical sources.

Class of Dietary Antioxidants	Subclass	Name	Sources
**Vitamins**	Vitamin A	α-Carotene	Carrots, tuna, butternut squash, sweet potato, spinach, cantaloupe, lettuce, etc.
		β-Carotene	
	Vitamin C	Ascorbic acid	Citrus fruits, tomatoes, potatoes, papaya, peppers, kiwifruit, green leafy vegetables, etc.
	Vitamin E	α-Tocopherol	Sunflower seeds, almonds, avocados, spinach, butternut squash, kiwifruit, olive oil, shrimp, etc.
		β-Tocopherol	
**Bioflavonoids**	Flavonols	Quercetin	Apples, citrus fruits, green leafy vegetables, etc.
		Myricetin	Tomatoes, citrus fruits, nuts, berries, tea, red wine, etc.
	Flavones	Apigenin	Parsley, onions, citrus fruits, tea, chamomile, wheat sprouts, etc.
		Baicalein	Scutellaria baicalensis, oroxylum indicum, thyme, etc.
		Luteolin	Celery, thyme, green peppers, chamomile tea, etc.
	Flavonolols	Taxifolin	Milk thistle seeds, onions, tamarind seeds, etc.
	Flavan-3-ols	Catechin	Apples, berries, grape seeds, kiwi, etc.
		Epigallocatechin	Green tea, berries, kiwi, cherries, pears, etc.
	Flavonones	Hesperidin	Citrus fruits, grapefruit, tangerines, etc.
		Naringenin	Grapefruit, bergamot, citrus, cherries, tomatoes, etc.
	Anthocyanidins	Cyanidin	Berries, grapes, cherry, apples, pears, etc.
		Delphinidin	Berries, grapes, sweet potatoes, cabbage, etc.
	Isoflavones	Genistein	Soybeans, etc.
		Daidzein	Soybeans, etc.
**Carotenoids**	Carotenes	Lycopene	Tomatoes, carrots, watermelons, grapefruits, papayas, red fruits, etc.
		β-carotene	Kale, spinach, sweet potatoes, carrots, etc.
	Xanthophylls	Zeaxanthin	Paprika, corn, saffron, berries, etc.
		Lutein	Nasturtium, pot marigold, kale, dandelion, nasturtium, spinach, broccoli, peas, etc.
**Hydroxycinna-mates**	*O*-methylated forms	Ferulic acid	Bamboo shoots, angelica sinensis, ferula assa-foetida, brassica pekinensis, spinach, garlic, etc.
		Sinapic acid	Citrus fruits, berries, cereals, sunflower, etc.
	Dihydroxycinnamic acids	Caffeic acid	Coffee, turmeric, basil, thyme, oregano, sage, cabbage, etc.
	Monohydroxycinnamic acids	P-coumaric acid	Peanuts, navy beans, tomatoes, carrots, basil, garlic, etc.
**Other types**		Theaflavin	Black Tea.
		Theaflavin-3-gallate	Black Tea.
		Allicin	Garlic.
		Piperine	Peppers.
		Curcumin	Curcuma longa.

**Table 3:** A list of representative neurodegenerative diseases that are accompanied by aberrant production of ROS and/or mitochondrial dysfunction.

Neurodegenerative Disease	Aberrant Production of ROS	Defects in Mitophagy	Defects in Fission and Fusion	Other Mitochondrial Defects	References
Adrenoleukodystrophy	Redox imbalance	N/A	N/A	Defective mitochondrial biogenesis	[141]
Agenesis of the Corpus Callosum	N/A	N/A	N/A	Raised urinary hydroxyglutaric acid levels and Krebs metabolites	[142]
Alexander Disease	N/A	N/A	N/A	Increase of lactic acid; mitochondrial structural abnormalities; decreased cytochrome c oxidase activity	[143,144]
Alpers Disease	Redox imbalance	N/A	N/A	Complex IV dysfunction; deficient activity of mitochondrial DNA polymerase γ	[145]
Amyotrophic Lateral Sclerosis	Increased ROS production	Enhanced mitophagy	Reduced mitochondrial length and density	Disrupted anterograde axonal transport of mitochondria	[146,147,148,149,150]
Alzheimer’s Disease	Increased ROS production	N/A	Defects in mitochondrial dynamics	Decreased cytochrome c oxidase and ATP production	[151,152,153]
Ataxia Telangiectasia	Increased ROS production	Decreased mitophagy	Alterations in mitochondrial fusion or fission	Decreased intracellular ATP levels	[154]
Batten Disease	N/A	N/A	N/A	Accumulation of subunit c of ATP synthase; structural abnormalities of mitochondria	[155]
Charcot–Marie–Tooth Disease	N/A	N/A	Impaired mitochondrial fusion	Altered energy production via a mitochondrial complex I deficiency	[156,157]
Creutzfeldt Jakob Disease	Increased ROS production	N/A	N/A	Deficiency of all MRC complexes	[158]
Dementia with Lewy Bodies	Increased ROS production	N/A	N/A	Lower flux of tricarboxylic acid cycle and oxidative phosphorylation	[159]
Frontotemporal Dementia	N/A	Defects in mitochondrial dynamics	N/A	Disrupted ER-mitochondria signaling	[160,161]
Gerstmann Straussler Scheinker Disease	N/A	N/A	N/A	Induction of mitochondria-mediated apoptosis	[162]
Giant Axonal Neuropathy	N/A	N/A	N/A	Inhibition of mitochondrial motility	[163,164]
Huntington’s Disease	Increased ROS production	N/A	Imbalanced fission/fusion	Inhibition of mitochondrial protein import	[165,166,167]
Infantile Neuroaxonal Dystrophy	N/A	N/A	N/A	Reduced mitochondrial Ca^2+^ uptake rate and Ca^2+^ retention capacity	[168]
Kennedy’s Disease	Increased ROS production	N/A	N/A	depolarization of the mitochondrial membrane	[169]
Krabbe Disease	N/A	N/A	N/A	Mitochondrial morphological defects	[170]
Machado-Joseph Disease	Increased ROS production (induced by complex II inhibition)	N/A	N/A	Enhanced cell death upon mitochondrial complex II inhibition	[171]
Menkes Disease	Redox imbalance	N/A	N/A	N/A	[172]
Multiple Sclerosis	Increased ROS production	N/A	N/A	Reduced energy production in axonal mitochondria	[173]
Multiple System Atrophy	Increased ROS levels	Impaired mitophagic machinery	N/A	Reduced complex II activity; increased mitochondrial mass	[174,175]
Parkinson’s Disease	Increased ROS production	Impaired mitophagy	Imbalanced fission/fusion	Disruption of mitochondrial morphology; decreased ETC enzyme activities; impaired biogenesis; impaired mitochondrial trafficking	[16,176,177,178,179,180]
Rett Syndrome	Redox imbalance	N/A	N/A	Alterations in mitochondrial ultrastructure	[181]
Troyer Syndrome	N/A	N/A	N/A	Cytochrome c oxidase deficiency; decreased mitochondrial Ca^2+^ uptake capacity	[182]
Zellweger Syndrome	N/A	N/A	N/A	Alterations in mitochondrial ultrastructure; changes in the expression and activities of mitochondrial respiratory chain complexes; increase in the heterogeneity of the mitochondrial compartment	[183]

## References

[1] Hofer S.M., Berg S., Era P. (2003). Evaluating the interdependence of aging-related changes in visual and auditory acuity, balance, and cognitive functioning. Psychol. Aging.

[2] Mattson M.P., Magnus T. (2006). Ageing and neuronal vulnerability. Nat. Rev. Neurosci..

[3] Mink J.W., Blumenschine R.J., Adams D.B. (1981). Ratio of central nervous system to body metabolism in vertebrates: Its constancy and functional basis. Am. J. Physiol. Integr. Comp. Physiol..

[4] Misgeld T., Schwarz T.L. (2017). Mitostasis in Neurons: Maintaining Mitochondria in an Extended Cellular Architecture. Neuron.

[5] Schon E.A., Manfredi G. (2003). Neuronal degeneration and mitochondrial dysfunction. J. Clin. Investig..

[6] Zhao X.-Y., Lu M.-H., Yuan D.-J., Xu D.-E., Yao P.-P., Ji W.-L., Chen H., Liu W.-L., Yan C.-X., Xia Y.-Y. (2019). Mitochondrial Dysfunction in Neural Injury. Front. Neurosci..

[7] Tatsuta T., Langer T. (2008). Quality control of mitochondria: Protection against neurodegeneration and ageing. EMBO J..

[8] Youle R.J., Van Der Bliek A.M. (2012). Mitochondrial Fission, Fusion, and Stress. Science.

[9] Ravanelli S., Brave F.D., Hoppe T. (2020). Mitochondrial Quality Control Governed by Ubiquitin. Front. Cell Dev. Biol..

[10] Ge P., Dawson V.L., Dawson T.M. (2020). PINK1 and Parkin mitochondrial quality control: A source of regional vulnerability in Parkinson’s disease. Mol. Neurodegener..

[11] Favaro G., Romanello V., Varanita T., Desbats M.A., Morbidoni V., Tezze C., Albiero M., Canato M., Gherardi G., De Stefani D. (2019). DRP1-mediated mitochondrial shape controls calcium homeostasis and muscle mass. Nat. Commun..

[12] Rangaraju V., Lauterbach M., Schuman E.M. (2019). Spatially Stable Mitochondrial Compartments Fuel Local Translation during Plasticity. Cell.

[13] Huang M.L.-H., Chiang S., Kalinowski D.S., Bae D.-H., Sahni S., Richardson D.R. (2019). The Role of the Antioxidant Response in Mitochondrial Dysfunction in Degenerative Diseases: Cross-Talk between Antioxidant Defense, Autophagy, and Apoptosis. Oxidative Med. Cell. Longev..

[14] Van Houten B., Woshner V., Santos J.H. (2006). Role of mitochondrial DNA in toxic responses to oxidative stress. DNA Repair.

[15] Winklhofer K.F., Haass C. (2010). Mitochondrial dysfunction in Parkinson’s disease. Biochim. Biophys. Acta.

[16] Park J.-S., Davis R.L., Sue C.M. (2018). Mitochondrial Dysfunction in Parkinson’s Disease: New Mechanistic Insights and Therapeutic Perspectives. Curr. Neurol. Neurosci. Rep..

[17] Whitworth A.J., Pallanck L.J. (2009). The PINK1/Parkin pathway: A mitochondrial quality control system?. J. Bioenerg. Biomembr..

[18] Park J., Lee S.B., Lee S., Kim Y., Song S., Kim S., Bae E., Kim J., Shong M., Kim J.-M. (2006). Mitochondrial dysfunction in Drosophila PINK1 mutants is complemented by parkin. Nat. Cell Biol..

[19] Clark I.E., Dodson M.W., Jiang C., Cao J.H., Huh J.R., Seol J.H., Yoo S.J., Hay B.A., Guo M. (2006). Drosophila pink1 is required for mitochondrial function and interacts genetically with parkin. Nat. Cell Biol..

[20] Villalba R.M., Smith Y. (2017). Loss and remodeling of striatal dendritic spines in Parkinson’s disease: From homeostasis to maladaptive plasticity?. J. Neural Transm..

[21] Koppula S., Han I.-B., More S.V., Lim H.-W., Hong S.-M., Choi D.-K. (2012). Recent Updates in Redox Regulation and Free Radical Scavenging Effects by Herbal Products in Experimental Models of Parkinson’s Disease. Molecules.

[22] Carrera-Juliá S., Moreno M.L., Barrios C., Ortí J.E.D.L.R., Drehmer E. (2020). Antioxidant Alternatives in the Treatment of Amyotrophic Lateral Sclerosis: A Comprehensive Review. Front. Physiol..

[23] Feng Y., Wang X. (2012). Antioxidant Therapies for Alzheimer’s Disease. Oxid. Med. Cell. Longev..

[24] Phaniendra A., Jestadi D.B., Periyasamy L. (2015). Free Radicals: Properties, Sources, Targets, and Their Implication in Various Diseases. Indian J. Clin. Biochem..

[25] Mittler R. (2017). ROS Are Good. Trends Plant Sci..

[26] Starkov A.A. (2008). The Role of Mitochondria in Reactive Oxygen Species Metabolism and Signaling. Ann. N. Y. Acad. Sci..

[27] Forman H.J., Ursini F., Maiorino M. (2014). An overview of mechanisms of redox signaling. J. Mol. Cell. Cardiol..

[28] Redza-Dut M., Averill-Bates D.A. (2016). Activation of apoptosis signalling pathways by reactive oxygen species. Biochim. Biophys. Acta.

[29] Turpaev K.T. (2002). Reactive Oxygen Species and Regulation of Gene Expression. Biochemistry.

[30] Morgan M.J., Liu Z.-G. (2010). Crosstalk of reactive oxygen species and NF-κB signaling. Cell Res..

[31] He L., He T., Farrar S., Ji L., Liu T., Ma X. (2017). Antioxidants Maintain Cellular Redox Homeostasis by Elimination of Reactive Oxygen Species. Cell. Physiol. Biochem..

[32] Balasubramanian B., Pogozelski W.K., Tullius T.D. (1998). DNA strand breaking by the hydroxyl radical is governed by the accessible surface areas of the hydrogen atoms of the DNA backbone. Proc. Natl. Acad. Sci. USA.

[33] Du J., Gebicki J.M. (2004). Proteins are major initial cell targets of hydroxyl free radicals. Int. J. Biochem. Cell Biol..

[34] Ayala A., Muñoz M.F., Argüelles S. (2014). Lipid Peroxidation: Production, Metabolism, and Signaling Mechanisms of Malondialdehyde and 4-Hydroxy-2-Nonenal. Oxid. Med. Cell. Longev..

[35] Belarbi K., Cuvelier E., Destée A., Gressier B., Chartier-Harlin M.-C. (2017). NADPH oxidases in Parkinson’s disease: A systematic review. Mol. Neurodegener..

[36] Brandes R.P., Weissmann N., Schröder K. (2014). Nox family NADPH oxidases: Molecular mechanisms of activation. Free. Radic. Biol. Med..

[37] Murphy M.P. (2008). How mitochondria produce reactive oxygen species. Biochem. J..

[38] Zhao R., Jiang S., Zhang L., Yu Z. (2019). Mitochondrial electron transport chain, ROS generation and uncoupling (Review). Int. J. Mol. Med..

[39] Fukai T., Ushio-Fukai M. (2011). Superoxide Dismutases: Role in Redox Signaling, Vascular Function, and Diseases. Antioxidants Redox Signal..

[40] Zeeshan H.M.A., Lee G.H., Kim H.-R., Chae H.-J. (2016). Endoplasmic Reticulum Stress and Associated ROS. Int. J. Mol. Sci..

[41] Frederiks W.M., Vreeling-Sindelárová H. (2002). Ultrastructural localization of xanthine oxidoreductase activity in isolated rat liver cells. Acta Histochem..

[42] Ulfig A., Leichert L.I. (2020). The effects of neutrophil-generated hypochlorous acid and other hypohalous acids on host and pathogens. Cell. Mol. Life Sci..

[43] Andreyev A.Y., Kushnareva Y.E., Murphy A.N., Starkov A.A. (2015). Mitochondrial ROS metabolism: 10 Years later. Biochemistry.

[44] Guo C., Sun L., Chen X., Zhang D. (2013). Oxidative stress, mitochondrial damage and neurodegenerative diseases. Neural Regen. Res..

[45] Toescu E.C. (2005). Normal brain ageing: Models and mechanisms. Philos. Trans. R. Soc. B Biol. Sci..

[46] Stewart V.C., Heales S.J.R. (2003). Nitric oxide-induced mitochondrial dysfunction: Implications for neurodegeneration. Free Radic. Biol. Med..

[47] Fujii J., Ikeda Y. (2002). Advances in our understanding of peroxiredoxin, a multifunctional, mammalian redox protein. Redox Rep..

[48] Dunning S., Rehman A.U., Tiebosch M.H., Hannivoort R.A., Haijer F.W., Woudenberg J., Heuvel F.A.V.D., Buist-Homan M., Faber K.N., Moshage H. (2013). Glutathione and antioxidant enzymes serve complementary roles in protecting activated hepatic stellate cells against hydrogen peroxide-induced cell death. Biochim. Biophys. Acta Mol. Basis Dis..

[49] Bokare A.D., Choi W. (2014). Review of iron-free Fenton-like systems for activating H_2_O_2_ in advanced oxidation processes. J. Hazard. Mater..

[50] Ramana K.V., Srivastava S., Singhal S.S. (2014). Lipid Peroxidation Products in Human Health and Disease. Oxidative Med. Cell. Longev..

[51] Stolwijk J.M., Garje R., Sieren J.C., Buettner G.R., Zakharia Y. (2020). Understanding the Redox Biology of Selenium in the Search of Targeted Cancer Therapies. Antioxidants.

[52] Kozakiewicz M., Kornatowski M., Krzywińska O., Kędziora-Kornatowska K. (2019). Changes in the blood antioxidant defense of advanced age people. Clin. Interv. Aging.

[53] Luceri C., Bigagli E., Femia A.P., Caderni G., Giovannelli L., Lodovici M. (2018). Aging related changes in circulating reactive oxygen species (ROS) and protein carbonyls are indicative of liver oxidative injury. Toxicol. Rep..

[54] Lü J.-M., Lin P.H., Yao Q., Chen C. (2009). Chemical and molecular mechanisms of antioxidants: Experimental approaches and model systems. J. Cell. Mol. Med..

[55] Livrea M.A., Tesoriere L., Bongiorno A., Pintaudi A.M., Ciaccio M., Riccio A. (1995). Contribution of vitamin A to the oxidation resistance of human low density lipoproteins. Free Radic. Biol. Med..

[56] Niki E. (2014). Role of vitamin E as a lipid-soluble peroxyl radical scavenger: In vitro and in vivo evidence. Free Radic. Biol. Med..

[57] Hillstrom R.J., Yacapin-Ammons A.K., Lynch S.M. (2003). Vitamin C inhibits lipid oxidation in human HDL. J. Nutr..

[58] Young A.J., Lowe G.M. (2018). Carotenoids—Antioxidant Properties. Antioxidants.

[59] Andreasen M.F., Landbo A.-K.R., Christensen L.P., Hansen A.Å., Meyer A.B.S. (2001). Antioxidant Effects of Phenolic Rye (*Secale cereale* L.) Extracts, Monomeric Hydroxycinnamates, and Ferulic Acid Dehydrodimers on Human Low-Density Lipoproteins. J. Agric. Food Chem..

[60] Birringer M. (2011). Hormetics: Dietary Triggers of an Adaptive Stress Response. Pharm. Res..

[61] Ježek P., Hlavatá L. (2005). Mitochondria in homeostasis of reactive oxygen species in cell, tissues, and organism. Int. J. Biochem. Cell Biol..

[62] Mailloux R.J. (2018). Mitochondrial Antioxidants and the Maintenance of Cellular Hydrogen Peroxide Levels. Oxidative Med. Cell. Longev..

[63] Guo J.D., Zhao X., Li Y., Li G.R., Liu X.L. (2018). Damage to dopaminergic neurons by oxidative stress in Parkinson’s disease (Review). Int. J. Mol. Med..

[64] Simon D.K., Tanner C.M., Brundin P. (2020). Parkinson Disease Epidemiology, Pathology, Genetics, and Pathophysiology. Clin. Geriatr. Med..

[65] Poewe W., Seppi K., Tanner C.M., Halliday G.M., Brundin P., Volkmann J., Schrag A., Lang E.A. (2017). Parkinson disease. Nat. Rev. Dis. Primers.

[66] Dias V., Junn E., Mouradian M.M. (2013). The role of oxidative stress in Parkinson’s disease. J. Parkinsons Dis..

[67] Fukushima T., Gao T., Tawara T., Hojo N., Isobe A., Yamane Y. (1997). Inhibitory effect of nicotinamide to paraquat toxicity and the reaction site on complex I. Arch. Toxicol..

[68] Perier C., Bové J., Vila M., Przedborski S. (2003). The rotenone model of Parkinson’s disease. Trends Neurosci..

[69] Callio J., Oury T.D., Chu C.T. (2005). Manganese superoxide dismutase protects against 6-hydroxydopamine injury in mouse brains. J. Biol. Chem..

[70] Richardson J.R., Quan Y., Sherer T.B., Greenamyre J.T., Miller G.W. (2005). Paraquat Neurotoxicity is Distinct from that of MPTP and Rotenone. Toxicol. Sci..

[71] Lambert A.J., Brand M.D. (2004). Inhibitors of the Quinone-binding Site Allow Rapid Superoxide Production from Mitochondrial NADH:Ubiquinone Oxidoreductase (Complex I). J. Biol. Chem..

[72] Hatcher J.M., Pennell K.D., Miller G.W. (2008). Parkinson’s disease and pesticides: A toxicological perspective. Trends Pharmacol. Sci..

[73] Geisler S., Holmstrom K.M., Treis A., Skujat D., Springer W., Weber S.S., Fiesel F.C., Kahle P.J. (2010). The PINK1/Parkin-mediated mitophagy is compromised by PD-associated mutations. Autophagy.

[74] Sekine S., Wang C., Sideris D.P., Bunker E., Zhang Z., Youle R.J. (2019). Reciprocal Roles of Tom7 and OMA1 during Mitochondrial Import and Activation of PINK. Mol. Cell.

[75] Jin S.M., Lazarou M., Wang C., Kane L.A., Narendra D.P., Youle R.J. (2010). Mitochondrial membrane potential regulates PINK1 import and proteolytic destabilization by PARL. J. Cell Biol..

[76] Pickrell A.M., Youle R.J. (2015). The Roles of PINK1, Parkin, and Mitochondrial Fidelity in Parkinson’s Disease. Neuron.

[77] Shimura H., Hattori N., Kubo S.-I., Mizuno Y., Asakawa S., Minoshima S., Shimizu N., Iwai K., Chiba T., Tanaka K. (2000). Familial Parkinson disease gene product, parkin, is a ubiquitin-protein ligase. Nat. Genet..

[78] Chen Y., Ii G.W.D. (2013). PINK1-Phosphorylated Mitofusin 2 Is a Parkin Receptor for Culling Damaged Mitochondria. Science.

[79] Lawler J.M., Barnes W.S., Wu G., Song W., Demaree S. (2002). Direct Antioxidant Properties of Creatine. Biochem. Biophys. Res. Commun..

[80] Sestili P., Martinelli C., Colombo E., Barbieri E., Potenza L., Sartini S., Fimognari C. (2011). Creatine as an antioxidant. Amino Acids.

[81] Guidi C., Potenza L., Sestili P., Martinelli C., Guescini M., Stocchi L., Zeppa S., Polidori E., Annibalini G., Stocchi V. (2008). Differential effect of creatine on oxidatively-injured mitochondrial and nuclear DNA. Biochim. Biophys. Acta Gen. Subj..

[82] Zhang Z., Zheng L., Zhao Z., Shi J., Wang X., Huang J. (2014). Grape seed proanthocyanidins inhibit H_2_O_2_-induced osteoblastic MC3T3-E1 cell apoptosis via ameliorating H_2_O_2_-induced mitochondrial dysfunction. J. Toxicol. Sci..

[83] Zhang X., Du L., Zhang W., Yang Y., Zhou Q., Du G.-H. (2017). Therapeutic effects of baicalein on rotenone-induced Parkinson’s disease through protecting mitochondrial function and biogenesis. Sci. Rep..

[84] Tamilselvam K., Braidy N., Manivasagam T., Essa M.M., Prasad N.R., Karthikeyan S., Thenmozhi A.J., Selvaraju S., Guillemin G.J. (2013). Neuroprotective Effects of Hesperidin, a Plant Flavanone, on Rotenone-Induced Oxidative Stress and Apoptosis in a Cellular Model for Parkinson’s Disease. Oxid. Med. Cell. Longev..

[85] Antunes M.S., Goes A.T., Boeira S.P., Prigol M., Jesse C.R. (2014). Protective effect of hesperidin in a model of Parkinson’s disease induced by 6-hydroxydopamine in aged mice. Nutrition.

[86] Yang F., Wolk A., Håkansson N., Pedersen N.L., Wirdefeldt K. (2017). Dietary antioxidants and risk of Parkinson’s disease in two population-based cohorts. Mov. Disord..

[87] Li Z., Wang P., Yu Z., Cong Y., Sun H., Zhang J., Zhang J., Sun C., Zhang Y., Ju X. (2015). The effect of creatine and coenzyme q10 combination therapy on mild cognitive impairment in Parkinson’s disease. Eur. Neurol..

[88] Zhang S., Hernan M., Chen H., Spiegelman D., Willett W., Ascherio A. (2002). Intakes of vitamins E and C, carotenoids, vitamin supplements, and PD risk. Neurology.

[89] Kieburtz K., Tilley B.C., Elm J.J., Babcock D., Hauser R., Ross G.W., Augustine A.H., Augustine E.U., Aminoff M.J., Writing Group for the NINDS Exploratory Trials in Parkinson Disease (NET-PD) Investigators (2015). Faculty Opinions recommendation of Effect of creatine monohydrate on clinical progression in patients with Parkinson disease: A randomized clinical trial. JAMA.

[90] Takeda A., Nyssen O.P., Syed A., Jansen E., Bueno-de-Mesquita B., Gallo V. (2014). Vitamin A and carotenoids and the risk of Parkinson’s disease: A systematic review and meta-analysis. Neuroepidemiology.

[91] Haas R.H. (2019). Mitochondrial Dysfunction in Aging and Diseases of Aging. Biology.

[92] Fearnley J.M., Lees A.J. (1991). Ageing and Parkinson’s disease: Substantia nigra regional selectivity. Brain.

[93] Ma S.Y., Röytt M., Collan Y., Rinne J.O. (1999). Unbiased morphometrical measurements show loss of pigmented nigral neurones with ageing. Neuropathol. Appl. Neurobiol..

[94] Ratner M., Farb D.H., Ozer J., Feldman R.G., Durso R. (2014). Younger age at onset of sporadic Parkinson’s disease among subjects occupationally exposed to metals and pesticides. Interdiscip. Toxicol..

[95] Nandipati S., Litvan I. (2016). Environmental Exposures and Parkinson’s Disease. Int. J. Environ. Res. Public Health.

[96] Hu G., Bidel S., Jousilahti P., Antikainen R., Toumilehto J. (2007). Coffee and tea consumption and the risk of Parkinson’s disease. Mov. Disord..

[97] Seidl S.E., Santiago J.A., Bilyk H., Potashkin J.A. (2014). The emerging role of nutrition in Parkinson’s disease. Front. Aging Neurosci..

[98] Morelli M., Carta A.R., Kachroo A., Schwarzschild M.A. (2010). Pathophysiological Roles for Purines: Adenosine, Caffeine and Urate.

[99] Sonsalla P.K., Wong L.-Y., Harris S.L., Richardson J.R., Khobahy I., Li W., Gadad B.S., German D.C. (2012). Delayed caffeine treatment prevents nigral dopamine neuron loss in a progressive rat model of Parkinson’s disease. Exp. Neurol..

[100] Singh S.S., Rai S.N., Birla H., Zahra W., Kumar G., Gedda M.R., Tiwari N., Patnaik R., Singh R.K., Singh S.P. (2018). Effect of Chlorogenic Acid Supplementation in MPTP-Intoxicated Mouse. Front. Pharmacol..

[101] Singh S.S., Rai S.N., Birla H., Zahra W., Rathore A.S., Dilnashin H., Singh R., Singh S.P. (2020). Neuroprotective Effect of Chlorogenic Acid on Mitochondrial Dysfunction-Mediated Apoptotic Death of DA Neurons in a Parkinsonian Mouse Model. Oxid. Med. Cell. Longev..

[102] Teraoka M., Nakaso K., Kusumoto C., Katano S., Tajima N., Yamashita A., Zushi T., Ito S., Matsura T. (2012). Cytoprotective effect of chlorogenic acid against α-synuclein-related toxicity in catecholaminergic PC12 cells. J. Clin. Biochem. Nutr..

[103] Anandhan A., Tamilselvam K., Radhiga T., Rao S., Essa M.M., Manivasagam T. (2012). Theaflavin, a black tea polyphenol, protects nigral dopaminergic neurons against chronic MPTP/probenecid induced Parkinson’s disease. Brain Res..

[104] Levites Y., Weinreb O., Maor G., Youdim M.B.H., Mandel S. (2001). Green tea polyphenol (-)-epigallocatechin-3-gallate prevents N-methyl-4-phenyl-1,2,3,6-tetrahydropyridine-induced dopaminergic neurodegeneration. J. Neurochem..

[105] Choe E., Min D.B. (2006). Chemistry and Reactions of Reactive Oxygen Species in Foods. Crit. Rev. Food Sci. Nutr..

[106] Miranda-Díaz A.G., García-Sánchez A., Cardona-Muñoz E.G. (2020). Foods with Potential Prooxidant and Antioxidant Effects Involved in Parkinson’s Disease. Oxid. Med. Cell. Longev..

[107] Kyrozis A., Ghika A., Stathopoulos P., Vassilopoulos D., Trichopoulos D., Trichopoulou A. (2013). Dietary and lifestyle variables in relation to incidence of Parkinson’s disease in Greece. Eur. J. Epidemiol..

[108] Park M., Ross G.W., Petrovitch H., White L.R., Masaki K.H., Nelson J.S., Tanner C.M., Curb J., Blanchette P.L., Abbott R.D. (2005). Consumption of milk and calcium in midlife and the future risk of Parkinson disease. Neurology.

[109] Chen H., Zhang S.M., Hernandez-Diaz S., Willett W.C., Ascherio A. (2002). Diet and Parkinson’s disease: A potential role of dairy products in men. Ann. Neurol..

[110] Morris J.K., Bomhoff G.L., Stanford J.A., Geiger P.C. (2010). Neurodegeneration in an animal model of Parkinson’s disease is exacerbated by a high-fat diet. Am. J. Physiol. Integr. Comp. Physiol..

[111] Bousquet M., St-Amour I., Vandal M., Julien P., Cicchetti F., Calon F. (2012). High-fat diet exacerbates MPTP-induced dopaminergic degeneration in mice. Neurobiol. Dis..

[112] Anderson C., Checkoway H., Franklin G.M., Beresford S., Smith-Weller T., Swanson P.D. (1999). Dietary factors in Parkinson’s disease: The role of food groups and specific foods. Mov. Disord..

[113] Dong J., Beard J.D., Umbach D.M., Park Y., Huang X., Blair A., Kamel F., Chen H. (2014). Dietary fat intake and risk for Parkinson’s disease. Mov. Disord..

[114] Chen H., Zhang S.M., Hernán M.A., Willett W.C., Ascherio A. (2003). Dietary intakes of fat and risk of Parkinson’s disease. Am. J. Epidemiol..

[115] Zoll J., Sánchez H., N’Guessan B., Ribera F., Lampert E., Bigard X., Serrurier B., Fortin D., Geny B., Veksler V. (2002). Physical activity changes the regulation of mitochondrial respiration in human skeletal muscle. J. Physiol..

[116] Lemez S., Baker J. (2015). Do Elite Athletes Live Longer? A Systematic Review of Mortality and Longevity in Elite Athletes. Sports Med. Open.

[117] Maron B.J., Thompson P.D. (2018). Longevity in elite athletes: The first 4-min milers. Lancet.

[118] Kirk-Sanchez N.J., McGough E.L. (2013). Physical exercise and cognitive performance in the elderly: Current perspectives. Clin. Interv. Aging.

[119] Petzinger G.M., Fisher B.E., McEwen S., Beeler J.A., Walsh J.P., Jakowec M.W. (2013). Exercise-enhanced neuroplasticity targeting motor and cognitive circuitry in Parkinson’s disease. Lancet Neurol..

[120] Wang H., Yuan G., Nanduri J., Boswell M., Katz D.M. (2006). Secretion of brain-derived neurotrophic factor from PC12 cells in response to oxidative stress requires autocrine dopamine signaling. J. Neurochem..

[121] Sleiman S.F., Henry J., Al-Haddad R., El Hayek L., Haidar E.A., Stringer T., Ulja D., Karuppagounder S.S., Holson E.B., Ratan R.R. (2016). Exercise promotes the expression of brain derived neurotrophic factor (BDNF) through the action of the ketone body β-hydroxybutyrate. eLife.

[122] Kim M.-W., Bang M.-S., Han T.-R., Ko Y.-J., Yoon B.-W., Kim J.-H., Kang L.-M., Lee K.-M., Kim M. (2005). Exercise increased BDNF and trkB in the contralateral hemisphere of the ischemic rat brain. Brain Res..

[123] Huang Y.Z., McNamara J.O. (2012). Neuroprotective Effects of Reactive Oxygen Species Mediated by BDNF-Independent Activation of TrkB. J. Neurosci..

[124] Liu P.Z., Nusslock R. (2018). Exercise-Mediated Neurogenesis in the Hippocampus via BDNF. Front. Neurosci..

[125] Mackay D.F., Russell E.R., Stewart K., MacLean J.A., Pell J.P., Stewart W. (2019). Neurodegenerative Disease Mortality among Former Professional Soccer Players. N. Engl. J. Med..

[126] Mahase E. (2019). Former footballers are more likely to die from neurodegenerative disease, study finds. BMJ.

[127] Hall E.D., Andrus P.K., Oostveen J.A., Fleck T.J., Gurney M.E. (1998). Relationship of oxygen radical-induced lipid peroxidative damage to disease onset and progression in a transgenic model of familial ALS. J. Neurosci. Res..

[128] Prasanthi J.R., Dasari B., Marwarha G., Larson T., Chen X., Geiger J.D., Ghribi O. (2010). Caffeine protects against oxidative stress and Alzheimer’s disease-like pathology in rabbit hippocampus induced by cholesterol-enriched diet. Free Radic. Biol. Med..

[129] Ishrat T., Parveen K., Khan M.B., Khuwaja G., Yousuf S., Ahmad A., Shrivastav P., Islam F. (2009). Selenium prevents cognitive decline and oxidative damage in rat model of streptozotocin-induced experimental dementia of Alzheimer’s type. Brain Res..

[130] Covarrubias-Pinto A., Moll P., Solís-Maldonado M., Acuña A.I., Riveros A., Miró M.P., Papic E., Beltrán F.A., Cepeda C., Concha I.I. (2015). Beyond the redox imbalance: Oxidative stress contributes to an impaired GLUT3 modulation in Huntington’s disease. Free Radic. Biol. Med..

[131] Wang T., Wang S., Wang X., Jiang H., Yang Y., Wang Y., Cheng J., Zhang C., Liang W., Feng H. (2018). Fisetin Exerts Antioxidant and Neuroprotective Effects in Multiple Mutant hSOD1 Models of Amyotrophic Lateral Sclerosis by Activating ERK. Neuroscience.

[132] Harraz M.M., Marden J.J., Zhou W., Zhang Y., Williams A.J., Sharov V.S., Nelson K., Luo M., Paulson H., Schöneich C. (2008). SOD1 mutations disrupt redox-sensitive Rac regulation of NADPH oxidase in a familial ALS model. J. Clin. Investig..

[133] West M., Mharte M., Caballos A., Floyd R.A., Grammas P., Gabbita S.P., Hamdheydari L., Mai T., Mou S., Pye Q.N. (2004). The arachidonic acid 5-lipoxygenase inhibitor nordihydroguaiaretic acid inhibits tumor necrosis factor alpha activation of microglia and extends survival of G93A-SOD1 transgenic mice. J. Neurochem..

[134] Hirohata M., Hasegawa K., Tsutsumi-Yasuhara S., Ohhashi Y., Ookoshi T., Ono K., Yamada M., Naiki H. (2007). The Anti-Amyloidogenic Effect Is Exerted against Alzheimer’s β-Amyloid Fibrils in Vitro by Preferential and Reversible Binding of Flavonoids to the Amyloid Fibril Structure†. Biochemistry.

[135] Lu J., Duan W., Guo Y., Jiang H., Li Z., Huang J., Hong K., Li C. (2012). Mitochondrial dysfunction in human TDP-43 transfected NSC34 cell lines and the protective effect of dimethoxy curcumin. Brain Res. Bull..

[136] Wang H., O’Reilly É.J., Weisskopf M.G., Logroscino G., McCullough M.L., Schatzkin A., Kolonel L.N., Ascherio A. (2011). Vitamin E Intake and Risk of Amyotrophic Lateral Sclerosis: A Pooled Analysis of Data From 5 Prospective Cohort Studies. Am. J. Epidemiol..

[137] Ano M.A.S., Ernesto C.H., Homas R.O.G.T., Klauber M.R., Chafer K.I.S., Rundman M.I.G., Oodbury J.O.W., Growdon J., Otman C.A.W.C., Feiffer E.R.P. (1997). A Controlled Trial of Selegiline, Alpha-Tocopherol, or Both as Treatment for Alzheimer’s Disease. N. Engl. J. Med..

[138] Ohlow M.J., Sohre S., Granold M., Schreckenberger M., Moosmann B. (2016). Why Have Clinical Trials of Antioxidants to Prevent Neurodegeneration Failed?—A Cellular Investigation of Novel Phenothiazine-Type Antioxidants Reveals Competing Objectives for Pharmaceutical Neuroprotection. Pharm. Res..

[139] Liguori I., Russo G., Curcio F., Bulli G., Aran L., Della-Morte D., Gargiulo G., Testa G., Cacciatore F., Bonaduce D. (2018). Oxidative stress, aging, and diseases. Clin. Interv. Aging.

[140] Dröge W. (2002). Free Radicals in the Physiological Control of Cell Function. Physiol. Rev..

[141] Baarine M., Beeson C., Singh A., Singh I. (2015). ABCD1 deletion-induced mitochondrial dysfunction is corrected by SAHA: Implication for adrenoleukodystrophy. J. Neurochem..

[142] Edvardson S., Porcelli V., Jalas C., Soiferman D., Kellner Y., Shaag A., Korman S.H., Pierri C.L., Scarcia P., Fraenkel N.D. (2013). Agenesis of corpus callosum and optic nerve hypoplasia due to mutations inSLC25A1encoding the mitochondrial citrate transporter. J. Med. Genet..

[143] Kang P.B., Hunter J.V., Kaye E.M. (2001). Lactic acid elevation in extramitochondrial childhood neurodegenerative diseases. J. Child Neurol..

[144] Cáceres-Marzal C., Vaquerizo J., Galán E., Fernández S. (2006). Early Mitochondrial Dysfunction in an Infant with Alexander Disease. Pediatr. Neurol..

[145] Naviaux R.K., Nyhan W.L., Barshop B.A., Poulton J., Markusic D., Karpinski N.C., Haas R.H. (1999). Mitochondrial DNA polymerase gamma deficiency and mtDNA depletion in a child with Alpers’ syndrome. Ann. Neurol..

[146] Hong K., Li Y., Duan W., Guo Y., Jiang H., Li W., Li C. (2012). Full-length TDP-43 and its C-terminal fragments activate mitophagy in NSC34 cell line. Neurosci. Lett..

[147] Braun R.J., Sommer C., Carmona-Gutierrez D., Khoury C.M., Ring J., Buettner S., Madeo F. (2011). Neurotoxic 43-kDa TAR DNA-binding Protein (TDP-43) Triggers Mitochondrion-dependent Programmed Cell Death in Yeast. J. Biol. Chem..

[148] Kruman I.I., Pedersen W.A., Springer J.E., Mattsonab M.P. (1999). ALS-Linked Cu/Zn–SOD Mutation Increases Vulnerability of Motor Neurons to Excitotoxicity by a Mechanism Involving Increased Oxidative Stress and Perturbed Calcium Homeostasis. Exp. Neurol..

[149] Wang W., Li L., Lin W.-L., Dickson D.W., Petrucelli L., Zhang T., Wang X. (2013). The ALS disease-associated mutant TDP-43 impairs mitochondrial dynamics and function in motor neurons. Hum. Mol. Genet..

[150] De Vos K.J., Mórotz G.M., Stoica R., Tudor E.L., Lau K.-F., Ackerley S., Warley A., Shaw C.E., Miller C.C. (2011). VAPB interacts with the mitochondrial protein PTPIP51 to regulate calcium homeostasis. Hum. Mol. Genet..

[151] Melov S., Adlard P.A., Morten K., Johnson F., Golden T.R., Hinerfeld D., Schilling B., Mavros C., Masters C.L., Volitakis I. (2007). Mitochondrial Oxidative Stress Causes Hyperphosphorylation of Tau. PLoS ONE.

[152] Trushina E., Nemutlu E., Zhang S., Christensen T., Camp J., Mesa J., Siddiqui A., Tamura Y., Sesaki H., Wengenack T.M. (2012). Defects in Mitochondrial Dynamics and Metabolomic Signatures of Evolving Energetic Stress in Mouse Models of Familial Alzheimer’s Disease. PLoS ONE.

[153] Reddy P.H. (2011). Abnormal tau, mitochondrial dysfunction, impaired axonal transport of mitochondria, and synaptic deprivation in Alzheimer’s disease. Brain Res..

[154] Valentin-Vega Y.A., MacLean K.H., Tait-Mulder J., Milasta S., Steeves M., Dorsey F.C., Cleveland J.L., Green D.R., Kastan M.B. (2012). Mitochondrial dysfunction in ataxia-telangiectasia. Blood.

[155] Jolly R.D. (2002). Mitochondrial dysfunction in the neuronal ceroid-lipofuscinoses (Batten disease). Neurochem. Int..

[156] Amiott E.A., Lott P.C., Soto J., Kang P.B., McCaffery J.M., DiMauro S., Abel E.D., Flanigan K.M., Lawson V.H., Shaw J.M. (2008). Mitochondrial fusion and function in Charcot–Marie–Tooth type 2A patient fibroblasts with mitofusin 2 mutations. Exp. Neurol..

[157] Cassereau J., Chevrollier A., Gueguen N., Desquiret-Dumas V., Verny C., Nicolas G., Dubas F., Amati-Bonneau P., Reynier P., Bonneau D. (2011). Mitochondrial dysfunction and pathophysiology of Charcot–Marie–Tooth disease involving GDAP1 mutations. Exp. Neurol..

[158] Zhang J., Zhang Z.-X., Du P.-C., Zhou W., Wu S.-D., Wang Q.-L., Chen C., Shi Q., Chen C., Gao C. (2014). Analyses of the mitochondrial mutations in the Chinese patients with sporadic Creutzfeldt–Jakob disease. Eur. J. Hum. Genet..

[159] Rajkumar A.P., Bidkhori G., Shoaie S., Clarke E., Morrin H., Hye A., Williams G., Ballard C., Francis P.T., Aarsland D. (2019). Postmortem Cortical Transcriptomics of Lewy Body Dementia Reveal Mitochondrial Dysfunction and Lack of Neuroinflammation. Am. J. Geriatr. Psychiatr..

[160] Choi S.Y., Lopez-Gonzalez R., Krishnan G., Phillips H.L., Li A.N., Seeley W.W., Yao W.-D., Almeida S., Gao F.-B. (2019). C9ORF72-ALS/FTD-associated poly(GR) binds Atp5a1 and compromises mitochondrial function in vivo. Nat. Neurosci..

[161] Lau D.H.W., Hartopp N., Welsh N.J., Mueller S., Glennon E.B., Mórotz G.M., Annibali A., Gomez-Suaga P., Stoica R., Paillusson S. (2018). Disruption of ER−mitochondria signalling in fronto-temporal dementia and related amyotrophic lateral sclerosis. Cell Death Dis..

[162] Hachiya N.S., Watanabe K., Kawabata M.Y., Jozuka A., Kozuka Y., Sakasegawa Y., Kaneko K. (2005). Prion protein with Y145STOP mutation induces mitochondria-mediated apoptosis and PrP-containing deposits in vitro. Biochem. Biophys. Res. Commun..

[163] Israeli E., Dryanovski D.I., Schumacker P.T., Chandel N.S., Singer J.D., Julien J.P., Goldman R.D., Opal P. (2016). Intermediate filament aggregates cause mitochondrial dysmotility and increase energy demands in giant axonal neuropathy. Hum. Mol. Genet..

[164] Lowery J., Jain N., Kuczmarski E.R., Mahammad S., Goldman A., Gelfand V.I., Opal P., Goldman R.D. (2016). Abnormal intermediate filament organization alters mitochondrial motility in giant axonal neuropathy fibroblasts. Mol. Biol. Cell.

[165] Kim J., Moody J.P., Edgerly C.K., Bordiuk O.L., Cormier K., Smith K., Beal M.F., Ferrante R.J. (2010). Mitochondrial loss, dysfunction and altered dynamics in Huntington’s disease. Hum. Mol. Genet..

[166] Yano H., Baranov S.V., Baranova O.V., Kim J., Pan Y., Yablonska S., Carlisle D.L., Ferrante R.J., Kim A.H., Friedlander R.M. (2014). Inhibition of mitochondrial protein import by mutant huntingtin. Nat. Neurosci..

[167] Shirendeb U., Reddy A.P., Manczak M., Calkins M.J., Mao P., Tagle D.A., Reddy P.H. (2011). Abnormal mitochondrial dynamics, mitochondrial loss and mutant huntingtin oligomers in Huntington’s disease: Implications for selective neuronal damage. Hum. Mol. Genet..

[168] Strokin M., Reiser G. (2016). Mitochondria from a mouse model of the human infantile neuroaxonal dystrophy (INAD) with genetic defects in VIA iPLA 2 have disturbed Ca 2+ regulation with reduction in Ca 2+ capacity. Neurochem. Int..

[169] Ranganathan S., Harmison G.G., Meyertholen K., Pennuto M., Burnett B.G., Fischbeck K.H. (2008). Mitochondrial abnormalities in spinal and bulbar muscular atrophy. Hum. Mol. Genet..

[170] Lim S.M., Choi B.-O., Oh S.-I., Choi W.J., Oh K.-W., Nahm M., Xue Y., Choi J.H., Choi J.Y., Kim Y.-E. (2016). Patient fibroblasts-derived induced neurons demonstrate autonomous neuronal defects in adult-onset Krabbe disease. Oncotarget.

[171] Laço M.N., Oliveira C.R., Paulson H.L., Rego A.C. (2012). Compromised mitochondrial complex II in models of Machado–Joseph disease. Biochim. Biophys. Acta Mol. Basis Dis..

[172] Bhattacharjee A., Yang H., Duffy M., Robinson E., Conrad-Antoville A., Lu Y.-W., Capps T., Braiterman L., Wolfgang M., Murphy M.P. (2016). The Activity of Menkes Disease Protein ATP7A Is Essential for Redox Balance in Mitochondria*. J. Biol. Chem..

[173] Witte M.E., Nijland P.G., Drexhage J.A.R., Gerritsen W., Geerts D., Hof B.V.H., Reijerkerk A., De Vries H.E., Van Der Valk P., Van Horssen J. (2012). Reduced expression of PGC-1α partly underlies mitochondrial changes and correlates with neuronal loss in multiple sclerosis cortex. Acta Neuropathol..

[174] Farley P.J. (1986). Hospital and ambulatory services for selected illnesses. Heal. Serv. Res..

[175] Compagnoni G.M., Kleiner G., Bordoni A., Fortunato F., Ronchi D., Salani S., Guida M., Corti C., Pichler I., Bergamini C. (2018). Mitochondrial dysfunction in fibroblasts of Multiple System Atrophy. Biochim. Biophys. Acta Mol. Basis Dis..

[176] Bose A., Beal M.F. (2016). Mitochondrial dysfunction in Parkinson’s disease. J. Neurochem..

[177] Rocha E.M., De Miranda B., Sanders L.H. (2018). Alpha-synuclein: Pathology, mitochondrial dysfunction and neuroinflammation in Parkinson’s disease. Neurobiol. Dis..

[178] Subramaniam S.R., Chesselet M.F. (2013). Mitochondrial dysfunction and oxidative stress in Parkinson’s disease. Prog. Neurobiol..

[179] Larsen S.B., Hanss Z., Krüger R. (2018). The genetic architecture of mitochondrial dysfunction in Parkinson’s disease. Cell Tissue Res..

[180] Ryan B.J., Hoek S., Fon E.A., Wade-Martis R. (2015). Mitochondrial dysfunction and mitophagy in Parkinson’s: From familial to sporadic disease. Trends Biochem. Sci..

[181] Jagtap S., Thanos J.M., Fu T., Wang J., LaLonde J., O’Dial T., Feiglin A., Chen J., Kohane I.S., Lee J.T. (2019). Aberrant mitochondrial function in patient-derived neural cells from CDKL5 deficiency disorder and Rett syndrome. Hum. Mol. Genet..

[182] Spiegel R., Soiferman D., Shaag A., Shalev S., Elpeleg O., Saada A., Baumgartner M.R., Patterson M., Rahman S., Peters V. (2016). Novel Homozygous Missense Mutation in SPG20 Gene Results in Troyer Syndrome Associated with Mitochondrial Cytochrome c Oxidase Deficiency. JIMD Rep..

[183] Baumgart E., Vanhorebeek I., Grabenbauer M., Borgers M., Declercq P.E., Fahimi H.D., Baes M. (2001). Mitochondrial Alterations Caused by Defective Peroxisomal Biogenesis in a Mouse Model for Zellweger Syndrome (PEX5 Knockout Mouse). Am. J. Pathol..

[184] Oyewole A.O., Birch-Machin M.A. (2015). Mitochondria-targeted antioxidants. FASEB J..

[185] Jauslin M.L., Meier T., Smith R.A.J., Murphy P.M. (2003). Mitochondria-targeted antioxidants protect Friedreich Ataxia fibroblasts from endogenous oxidative stress more effectively than untargeted antioxidants. FASEB J..

[186] Genrikhs E.E., Stelmashook E.V., Popova O.V., Kapay N.A., Korshunova G.A., Sumbatyan N.V., Skrebitsky V.G., Skulachev V.P., Isaev N.K. (2015). Mitochondria-targeted antioxidant SkQT1 decreases trauma-induced neurological deficit in rat and prevents amyloid-β-induced impairment of long-term potentiation in rat hippocampal slices. J. Drug Target..

[187] Snow B.J., Rolfe F.L., Lockhart M.M., Frampton C.M., O’Sullivan J.D., Fung V., Smith R.A.J., Murphy M.P., Taylor K.M. (2010). A double-blind, placebo-controlled study to assess the mitochondria-targeted antioxidant MitoQ as a disease-modifying therapy in Parkinson’s disease. Mov. Disord..

[188] Zinovkin R.A. (2019). Mitochondria-Targeted Drugs. Curr. Mol. Pharmacol..

[189] Stefanova N.A., Muraleva N.A., Maksimova K.Y., Rudnitskaya E.A., Kiseleva E., Telegina D.V., Kolosova N.G. (2016). An antioxidant specifically targeting mitochondria delays progression of Alzheimer’s disease-like pathology. Aging.

[190] Pavshintsev V.V., Podshivalova L.S., Frolova O.Y., Belopolskaya M.V., Averina O.A., Kushnir E.A., Marmiy N.V., Lovat M.L. (2017). Effects of mitochondrial antioxidant SkQ1 on biochemical and behavioral parameters in a Parkinsonism model in mice. Biochemistry.

[191] Petrov A., Perekhvatova N., Skulachev M., Stein L., Ousler G.W. (2016). SkQ1 Ophthalmic Solution for Dry Eye Treatment: Results of a Phase 2 Safety and Efficacy Clinical Study in the Environment and During Challenge in the Controlled Adverse Environment Model. Adv. Ther..

[192] Seen S., Tong L. (2017). Dry eye disease and oxidative stress. Acta Ophthalmol..

[193] Pizzino G., Irrera N., Cucinotta M., Pallio G., Mannino F., Arcoraci V., Squadrito F., Altavilla D., Bitto A. (2017). Oxidative Stress: Harms and Benefits for Human Health. Oxidative Med. Cell. Longev..

[194] Keum Y.-S., Jeong W.-S., Kong A.T. (2004). Chemoprevention by isothiocyanates and their underlying molecular signaling mechanisms. Mutat. Res. Mol. Mech. Mutagen..

[195] Surh Y.J., Kundu J.K., Na H.K., Lee J.S. (2005). Redox-sensitive transcription factors as prime targets for chemoprevention with anti-inflammatory and antioxidative phytochemicals. J. Nutr..

[196] Son T.G., Camandola S., Mattson M.P. (2008). Hormetic Dietary Phytochemicals. NeuroMol. Med..

[197] Köhle C., Bock K.W. (2006). Activation of coupled Ah receptor and Nrf2 gene batteries by dietary phytochemicals in relation to chemoprevention. Biochem. Pharmacol..

[198] Li W., Kong A.-N. (2009). Molecular mechanisms of Nrf2-mediated antioxidant response. Mol. Carcinog..

[199] Muller M., Banning A., Brigelius-Flohé R., Kipp A. (2010). Nrf2 target genes are induced under marginal selenium-deficiency. Genes Nutr..

